# Fluidic integrated 3D bioprinting system to sustain cell viability towards larynx fabrication

**DOI:** 10.1002/btm2.10423

**Published:** 2022-10-20

**Authors:** Hae Sang Park, Ji Seung Lee, Chang‐Beom Kim, Kwang‐Ho Lee, In‐Sun Hong, Harry Jung, Hanna Lee, Young Jin Lee, Olatunji Ajiteru, Md Tipu Sultan, Ok Joo Lee, Soon Hee Kim, Chan Hum Park

**Affiliations:** ^1^ Department of Otorhinolaryngology–Head and Neck Surgery, Chuncheon Sacred Heart Hospital, College of Medicine Hallym University Chuncheon Republic of Korea; ^2^ Nano‐Bio Regenerative Medical Institute, School of Medicine Hallym University Chuncheon Republic of Korea; ^3^ Institute of New Frontier Research Team Hallym University, Hallym Clinical and Translation Science Institute Chuncheon Republic of Korea; ^4^ Intelligent Robot Research Team Electronics and Telecommunications Research Institute Daejeon Republic of Korea; ^5^ Department of Advanced Materials Science and Engineering, College of Engineering Kangwon National University Chuncheon Republic of Korea; ^6^ Department of Molecular Medicine, School of Medicine Gachon University Incheon Republic of Korea

**Keywords:** artificial larynx, laryngectomy, three‐dimensional bioprinting, tissue engineering

## Abstract

Herein, we report the first study to create a three‐dimensional (3D) bioprinted artificial larynx for whole‐laryngeal replacement. Our 3D bio‐printed larynx was generated using extrusion‐based 3D bioprinter with rabbit's chondrocyte‐laden gelatin methacryloyl (GelMA)/glycidyl‐methacrylated hyaluronic acid (GMHA) hybrid bioink. We used a polycaprolactone (PCL) outer framework incorporated with pores to achieve the structural strength of printed constructs, as well as to provide a suitable microenvironment to support printed cells. Notably, we established a novel fluidics supply (FS) system that simultaneously supplies basal medium together with a 3D bioprinting process, thereby improving cell survival during the printing process. Our results showed that the FS system enhanced post‐printing cell viability, which enabled the generation of a large‐scale cell‐laden artificial laryngeal framework. Additionally, the incorporation of the PCL outer framework with pores and inner hydrogel provides structural stability and sufficient nutrient/oxygen transport. An animal study confirmed that the transplanted 3D bio‐larynx successfully maintained the airway. With further development, our new strategy holds great potential for fabricating human‐scale larynxes with in vivo‐like biological functions for laryngectomy patients.

## 1 INTRODUCTION

Three‐dimensional (3D) bioprinting involves the fabrication of cell‐laden bioinks into functional tissue/organ constructs using 3D digital models for human tissue and organ regeneration.^1^, ^2^ Recent advances in tissue engineering have enabled 3D bioprinting using various biocompatible materials, including living cells, thereby making the product clinically applicable. Human laryngeal allotransplantation has long been anticipated as a therapeutic option for improving the quality of life (breathing without tracheostoma, normal swallowing, and speaking) of laryngectomized patients.^3^ However, the requirement for post‐transplant immunosuppressive treatment is an ethical concern for the larynx, which is a non‐vital organ. Recently, decellularized matrices have also been considered as an alternative option in several studies.^4^, ^5^, ^6^ Although the ECM scaffold tends to facilitate tissue regeneration, its preparation method (decellularization approach) can substantially alter the biomechanical properties of the resulting scaffold, which compromises its ability to provide mechanical support during the regeneration process.^6^, ^7^ Moreover, the major disadvantage of the allotransplantation/decellularization method is the difficulty in obtaining donor tissues/organs. From this point of view, 3D bioprinting is a potential technology that could solve this problem, despite its technical limitations. Despite significant advances in 3D bio‐printing technology, it remains a challenge to print bioinks to achieve long‐term stable structures and maintain high cell survival rates after printing.

In this study, we aimed to establish an optimized multimaterial bioprinting methodology for a microextrusion‐based 3D bioprinted larynx with chondrocyte‐laden GelMA/glycidyl‐methacrylated hyaluronic acid (GMHA) bioink. Multimaterial bioprinting is a promising technology integrating multimaterial setups into bioprinting systems for fabricating functional, mechanically stable tissue constructs.^8^ For generating biocompatible, multi‐cellular, macroscale structure with structural integrity, several studies have suggested various methods, including surface‐tension assisted 3D printing, microfluidic systems which facilitate the assembly of 3D tissue models.^9^, ^10^, ^11^ However, recent advances in multimaterial bioprinting have limited use in the regeneration of simple anatomical structure. In particular, the field of laryngeal tissue engineering is still in its early stages compared to other organ engineering techniques. This is because the larynx has a very complex structure and various functions compared to other organs. Recently, 3D bioprinting of the larynx using a gelatin methacryloyl (GelMA) bioink blended with decellularized extracellular matrix microparticles has been reported. This method enables the manufacturing of complex laryngeal geometries without requiring support or suspension gels.^12^

In our previous study, we developed photocurable GelMA/GMHA hybrid bioink for cartilage regeneration with tonsil‐derived mesenchymal stem cells (MSCs).^13^ It has been suggested as an optimal bioink with excellent cell viability, mechanical properties, rheological properties, and printability. The success of this study led us to hypothesize that the GelMA/GMHA bioink is a suitable bioink for 3D bioprinting of the human larynx, which is composed of six cartilages (three unpaired and three paired) that form its skeleton. For successful larynx bioprinting, three major strategies were used in this study: (1) mechanical stability was achieved by printing chondrocyte‐laden bioinks together with polycaprolactone (PCL); (2) to achieve high cell viability, 200–500 μm sized pores were created in the PCL framework; (3) in particular, we established a novel fluidics supply (FS) system that simultaneously supplies basal medium to the suspension bath during the 3D bioprinting process, thereby improving cell survival during the printing process. We hypothesized that the FS system would decrease cell damage by lowering the temperature of the extruded PCL, and preventing dehydration of the bioink during extended printing time, over 60 min.

To the best of our knowledge, this is the first study to generate a 3D bio‐printed artificial larynx using multimaterial bioprinting and evaluate its applicability.

## 2 MATERIALS AND METHODS

### 2.1 Isolation and culture of rabbit auricular chondrocytes

Auricular cartilage was obtained from a New Zealand rabbit (15‐ to 20‐week‐old, weight 3.5–4.5 kg). The cartilage was chopped into approximately 2‐mm pieces and digested with collagenase type II (CAT no. LS004177, Worthington Biochemical Corporation, Lakewood, NJ, USA) in DMEM/F12 (free serum, 1% A/A) (Gibco, Waltham, MA, USA) at 30% (v/v) for 48 h at 37°C, 5% CO_2_. Isolated chondrocytes were suspended and cultured in DMEM/F12 (free serum, A/A 1%) (Gibco, Waltham, MA, USA) supplemented with 10% bovine serum (Sigma‐Aldrich, St. Louis, MO, USA) and 1% A/A (antibiotics/antimycotics) in a 175‐T flask; the medium was renewed every 3 days.

### 2.2 ^1^H NMR spectroscopy

Gelatin, HA, GelMA, and GMHA (without lithium phenyl‐2,4,6 trimethylbenzoylphosphinate [LAP]) were dissolved in deuterium oxide (D_2_O) (Sigma‐Aldrich, St. Louis, MO, USA) for ^1^H NMR spectroscopy. Solution concentration was 0.2% (w/v) and each solution was measured using FT‐NMR 400 MHz spectrometer (JNM‐ECZ400s/L1, JEOL, Peabody, MA, USA) at room temperature.

### 2.3 Rheological and mechanical measurements

The rheological properties (viscosity and shear‐stress) of GelMA 7%/GMHA 5% bioink (G7H5) were measured at 24°C using a rotating rheometer (MCR102, Anton Paar, Ostfildern, Germany) operating in the vibration mode with a strain of 0.1% and frequency of 1 Hz. A total of 1 ml G7H5 bioink (without a photoinitiator, LAP) was placed on a 25‐mm plate to measure the viscosity and shear stress. Gelation point test was performed at 30°C. To measure viscoelasticity, 1 ml of G7H5 bioink (with LAP) was placed on a 24‐well plate using a 1‐cc syringe and cured for 20 s with a UV (365 nm) machine (USHIO, Tokyo, Japan). Subsequently, a disc of diameter 8 mm was created using an 8‐mm bipolar punch. For the mechanical test, G7H5 hydrogel, PCL, and PCL + G7H5 tube‐shaped constructs with 12‐mm outer diameter, 10.5‐mm inner diameter, and 8‐mm height were fabricated. The wall thickness (300 μm) and pore size (200–500 μm) of the PCL parts were designed to be the same as those of 3D‐bio larynx. The compressive strength was measured using a universal testing machine (QM100S, QMESYS, Gunpo, Korea) with a maximum load of 10 kgf at a crosshead speed of 5 mm/min. At least three specimens were used to measure their compressive strengths.

### 2.4 Cell proliferation test

GelMA (0.7 g), GMHA (0.5 g), 0.03% photoinitiator, and lithium phenyl‐2,4,6‐trimethylbenzoylphosphinate (LAP) were mixed in 10 ml of DMEM/F12 (free serum, A/A 1%), and then 100 μl of this mixture was cured by UV. The cured mixture was incubated in DMEM/F12 (free serum, 1% A/A) for 3 days, and supernatant was collected for cell proliferation tests. Rabbit chondrocytes (1 × 10^4^ cells/ml) were seeded in 24‐well plates and grown for 3 days in either supernatant of DMEM/F12 (free serum, A/A 1%) or the supernatant of G7H5 dissolved media. Cell proliferation was assessed using the IN CELL analysis 2200 imaging system (Cytiva, Chicago, IL, USA). Analysis was performed using the IN CELL Developer program (Cytiva, Chicago, IL, USA). All experiments were conducted three times.

### 2.5 Preparation of bioink and 3D bioprinting process

**FIGURE 1:**
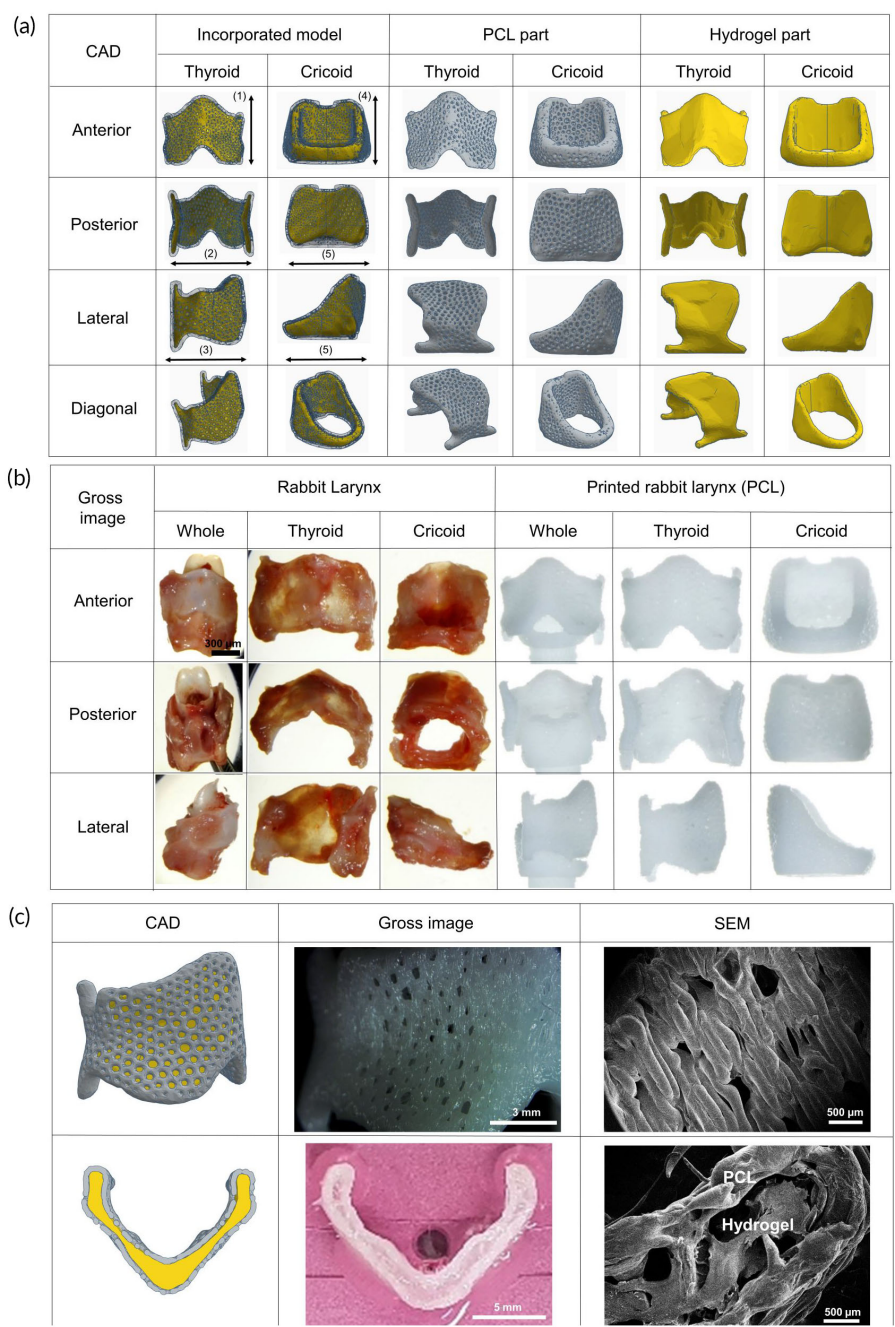
3D CAD modeling and printing of the rabbit larynx. (a) PCL model and hydrogel model were incorporated into the 3D CAD model of the 3D‐bio larynx. (b) Larynx of 20‐week‐old rabbits was harvested and observed for generating the larynx scaffold. (c) Chondrocytes‐laden G7H5 bioink was bio‐printed into the space between PCL outer framework. PCL, polycaprolactone; G7H5 bioink, GelMA 7%/GMHA 5% bioink; (1) 11.5 mm; (2) 14.1 mm; (3) 9.2 mm; (4) 8.05 mm; (5) 10.91 mm; (6) 10.95 mm

A 3D larynx rabbit model was generated using commercial computer‐aided design (CAD) software (CADian3D®, version 2015, Intelli Korea, South Korea), which was designed using Meshmixer (AutoCAD software, Autodesk Inc., San Rafael, CA, USA), and the hydrogel part was created using Tinkercad (AutoCAD software, Autodesk Inc., San Rafael, CA, USA). The PCL framework, as the basic structure of the bioprinted larynx, was 3D‐printed based on the following process; PCL (Purasorb® PC 12, CorbionPurac, Amsterdam, The Netherlands) was placed in a heating tank at 68°C and extruded through a nozzle using 3D‐Discovery™ 3D bio printer (regenHu, Fribourg, Switzerland). The inner diameter of the nozzle was 200 μm and the printing feed rate was 7 mm/s. The support structures were not used during the printing process. The thyroid framework had the following dimensions: height = 11.5 mm; width = 14.1 mm; anteroposterior diameter = 9.2 mm; pore size = 200–500 μm; and wall thickness = 300 μm. The ring‐shaped cricoid framework had the following dimensions: height = 8.05 mm; width = 10.91 mm; anteroposterior diameter = 10.95 mm; pore size = 200–500 μm; and wall thickness = 300 μm (Figure 1a,b). Chondrocyte‐laden G7H5 bioink was bio‐printed into the space between PCL outer framework using different syringes (inner diameter of nozzle = 250 μm; printing feed rate = 4 mm/s; cartridge temperature = 20°C) (Figure 1c). No support or suspension gel was required in this 3D bioprinting process. The temperature of the printed PCL was measured using an FLIR ONE camera (FLIR ONE Pro‐Android, FLIR Systems Inc., Wilsonville, OR, USA).

**FIGURE 2:**
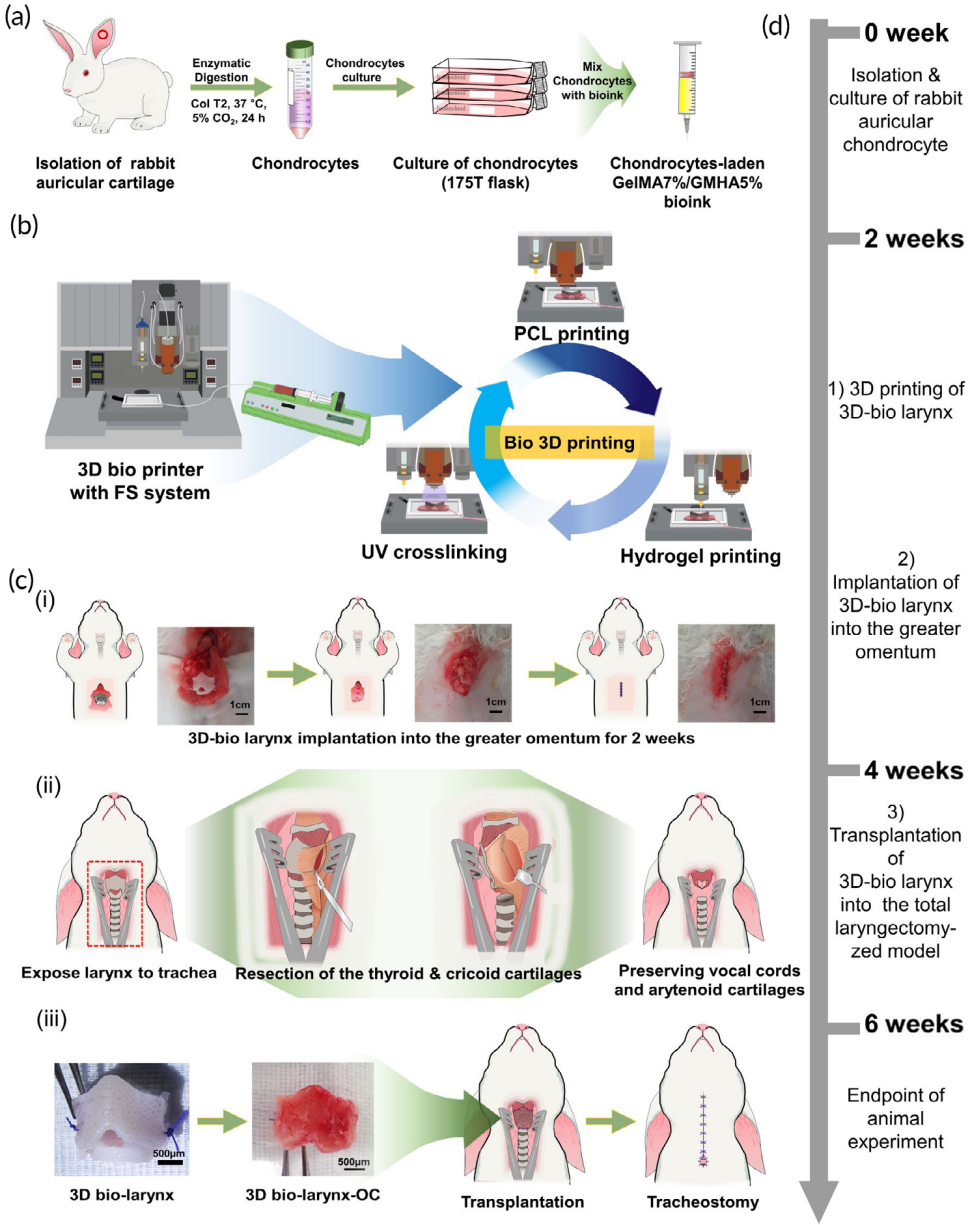
Schematic of larynx reconstruction. (a) Preparation of rabbit auricular chondrocytes‐laden GelMA7%/GMHA5% bioink. (b) 3D‐printing process using the fluidics supply system. (c) 3D‐bio larynx was implanted into the greater omentum for 2 weeks to (i) enable pre‐vascularization of the constructs; (ii) then, total laryngectomized rabbit model; (iii) received the transplanted pre‐vascularized construct. Col T2, Collagenase type 2; FS, fluidics supply; PCL, polycaprolactone; 3D bio‐larynx‐OC, 3D bio‐printed larynx after 2 weeks of omental culture. (d) Timeline of the experimental process

Chondrocytes‐loaded G7H5 bioink was prepared as described in a previous study.^13^ Briefly, for GelMA, 10% of A‐type skin gelatin (Sigma‐Aldrich, St. Louis, MO, USA) was dissolved in PBS, and stirred for 1 h at 60°C. Methacrylate anhydride (MA) (Sigma Aldrich, St. Louis, MO, USA) solution was added to the gelatin solution using a syringe pump at a rate of 0.4 ml/min until a 4% (v/v) concentration of MA in the gelatin solution was achieved. After completion of the reaction for 2 h, the solution was dialyzed with DW for 1 week in a 12–14 kDa dialysis tube. GelMA was obtained by freeze‐drying after dialysis and stored at −20°C. For GMHA, 1 g of HA (Sinochem, Qingdao, China, 1100 kDa) was stirred in PBS for 2 h. Then, 7.5 g of trimethylamine (Sigma‐Aldrich, St. Louis, MO, USA) and tetrabutyl ammonium bromide (Sigma‐Aldrich, St. Louis, MO, USA) were added and reacted for 30 min. Subsequently, 7.5 ml of glycidyl methacrylate (GM) (Sigma‐Aldrich, St. Louis, MO, USA) was added and allowed to react overnight. The GMHA solution was collected in 12–14 kDa rated dialyzer tubes and dialyzed at room temperature for 1 week, with DW replacement every 8 h. After dialysis, GMHA solution was collected to be frozen overnight at −80°C, and lyophilized. Freeze‐dried GMHA was stored at −80°C before reconstitution. The bioink used in the experiment was prepared by mixing 7% GelMA, 5% GMHA, 0.03% photoinitiator, and lithium phenyl‐2,4,6 trimethylbenzoylphosphinate (LAP) (Sigma‐Aldrich, St. Louis, MO, USA) (G7H5) in DMEM/F12. Chondrocytes were detached using 0.25% trypsin and 1 × 10^7^ cells/ml were mixed with the bioink solution (Figure 2a).

### 2.6 FS system

**FIGURE 3:**
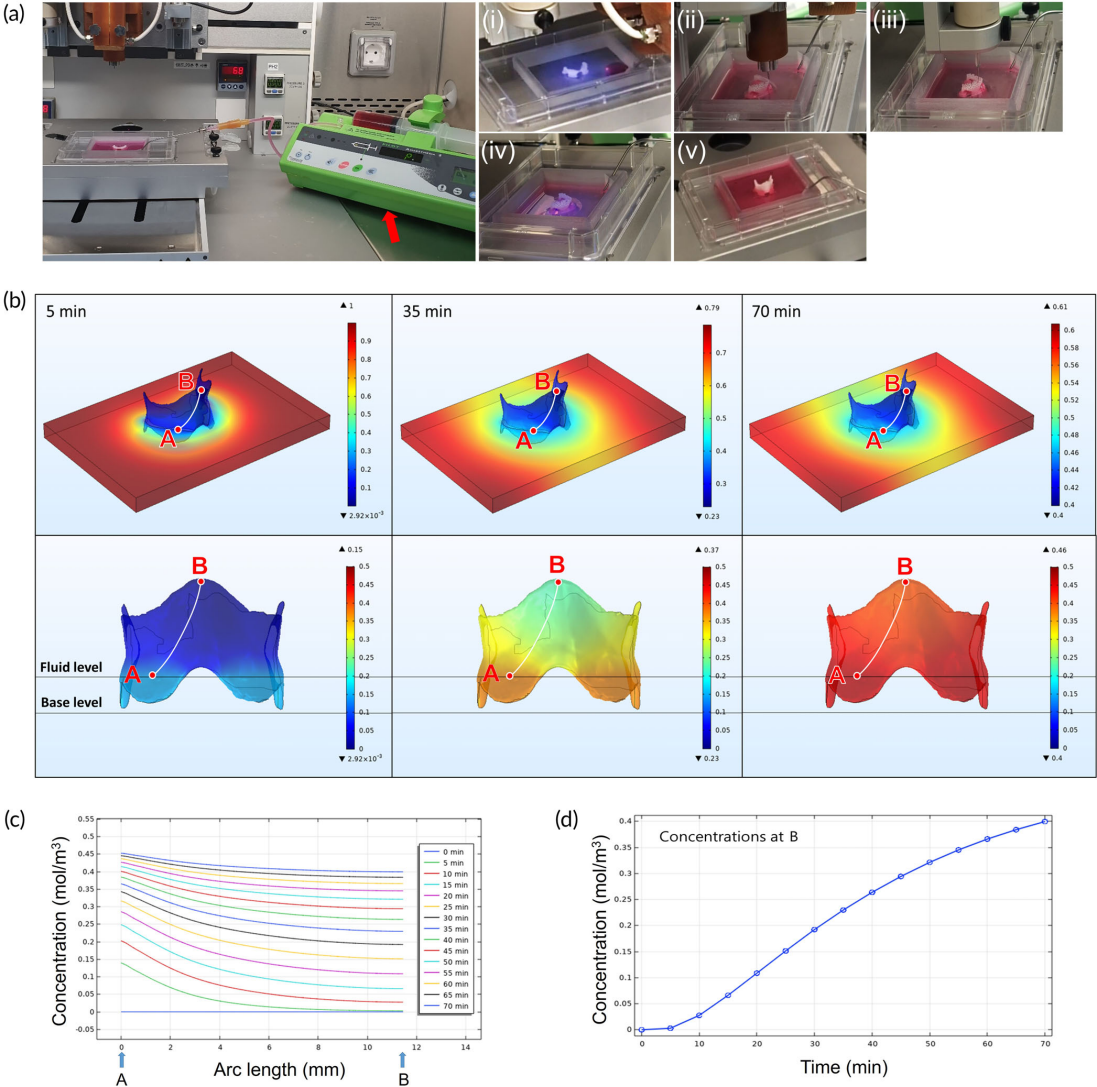
3D‐bio printing process with fluidics supply (FS) system and computational simulation. (a) The FS system consists of automatic syringe pump (red arrow), 50 ml syringe, and dispensing nozzle. (i) Fluidics supply system, (ii) PCL printing, (iii) G7H5 bioink printing, (iv) UV curing of the G7H5 bioink, and (v) 3D‐bio larynx (thyroid cartilage part). (b) Simulation images of glucose diffusion through the 3D printed larynx at 5, 35, and 70 min. (c) Graph of glucose molecules distribution across the length of an arc drawn from point A (inferior part of larynx) to point B (superior part of larynx) of (b) at regular intervals. (d) Graph of glucose molecule concentration versus time at point B (laryngeal prominence)

The FS system consisted of a syringe pump with a 50‐ml syringe (Fresenius Pilote Anesthesia 2, Codeo Medical, Chemin de la Morelle, France) and a dispensing nozzle (Figure 3a; Video S1). DMEM/F12 (free serum, A/A 1%) (Gibco, Waltham, MA, USA) or PBS was supplied to the suspension bath during the printing process via dispensing nozzle. The optimal supply amount and speed were calculated using the following computational model:

### 2.7 Computational modeling of the FS system

The diffusive transfer of glucose into the partially immersed 3D‐bio larynx in the medium was simulated computationally. A computational porous material model was applied to compare the concentration transport with the experimental results using effective transport properties, such as diffusion coefficient, dispersion coefficient, and porosity. A commercial finite element method (FEM)‐based simulation tool, COMSOL (version 5.4, COMSOL Inc., Burlington, MA, USA), was utilized for numerical analysis to evaluate the diffusive transfer of glucose into the larynx model of various hydrogels. A 3D porous model was defined through a simulation with effective porosity properties. In addition, to determine the temporal diffusive variation, a time‐dependent model was employed, and the governing equations were based on the Millington and Quirk diffusivity model, as follows:

(1) $$\frac{\partial c}{\partial t}+\nabla ∙\left[-\left(D_{d}+D_{e}\right)\right]\nabla c=0,$$

where *c* represents the glucose concentration (mol/m^3^) in the G7H5 bioink‐based laryngeal model, *D*_*d*_ is the dispersion coefficient (m^2^/s), and *D*_*e*_ is the diffusion coefficient (m^2^/s). Based on the mass transfer in porous media owing to the concentration gradient, only the diffusion and dispersion phenomena were analyzed in this simulation. In addition, no forced‐convection mechanism was considered in the medium domain. The relationship between diffusion and dispersion is defined as follows:

(2) $$D_{e}=\frac{\in_{p}}{\tau_{f}}D_{f},$$

where $\in_{p}$ denotes the porosity of the porous material, $\tau_{f}$ is the effective diffusivity and is equal to ${\in_{p}}^{-1/3}$, and *D*_*f*_ is the fluid diffusion coefficient (m^2^/s).

Figure 3b shows the 3D‐bio larynx simulation model (height 11.5 mm; width 14.1 mm). The larynx structure was assumed to be 3 mm and was immersed in the culture medium dish. Only the immersed parts of the 3D‐bio larynx were considered as the passage of the concentration transfer into the whole larynx structure. For a more accurate analysis, the full‐scale of the 3D‐bio larynx and culture medium domain were analyzed. The initial glucose concentration in the laryngeal domain was defined as a constant, $c\left(t_{0}\right)=14.0044\;\mathrm{mM}$. Two different boundary conditions were applied to the immersed and non‐immersed parts, based on the medium surface. The boundary condition at the non‐immersed surface of the 3D‐bio larynx was considered as no flux of medium through the surface, while the external flux was considered as the boundary condition at the immersed surface, which was not by forced convective flux but by diffusion due to the glucose concentration differences between the 3D‐bio larynx and medium domain as follows:

(3) $$-\left(D_{d}+D_{e}\right)\nabla c∙\vec{n}=k_{m}\left(c_{\mathrm{med}}-c\right),$$

where $\vec{n}$ denotes the normal vector, *k*_*m*_ is the mass transfer coefficient (m/s) in the 3D‐bio larynx, and *c*_med_ denotes the glucose concentration (mol/m^3^) in the culture medium domain. The culture medium containing the dish was modeled in the shape of a hexahedron with dimensions of 40 mm in length, 60 mm in width, and 4 mm in height, while the actual size of the dish used in the experiments was larger than that used in this simulation. Because the larynx structure was assumed to be 3 mm immersed in the culture medium, the boundary conditions of the immersed parts were defined as the external flux type with the mass transfer coefficient *k*_*m*_. The boundary conditions for the other surfaces, such as the top, bottom, and four side surfaces, were considered as no flux of the medium through the boundary. The interfaces between the medium and 3D‐bio larynx were assumed to have the same glucose concentration for both domains. The initial glucose concentration in the dish was set to $c_{\mathrm{med}}\left(t_{0}\right)=17.5055\;\mathrm{mM}$. For the meshing process, both the 3D‐bio larynx and medium domains were discretized into 3D‐tetrahedral meshes using a predefined fine element size with a maximum of 2 mm and a minimum of 300 μm. For high accuracy and rapid convergence of the results, the boundaries around the immersed volumes were discretized using the boundary layer properties with eight layers for both domains and a boundary layer stretching factor of 1.2. The maximum element size was 50 μm.

### 2.8 Cell viability by live‐and‐dead

Cytotoxicity tests were performed using a live/dead assay kit (Life Technologies, Carlsbad, CA, USA) according to the manufacturer's protocol. Images were captured using a K1‐fluo confocal laser scanning microscope (Nanoscope Systems, Daejeon, Korea).

### 2.9 Scanning electron microscope

A low‐vacuum SEM (JSM 6010 PLUS/LV, JEOL, Tokyo, Japan) was used to observe the morphology of the in vitro cultured 3D‐bio larynx, which was cultured in DMEM/F12 (free serum, A/A 1%) for 2 weeks (*n* = 3). The prepared scaffolds were cross‐sectioned and used for SEM imaging. The samples of each scaffold were coated with a thin 10‐nm layer of gold/palladium for 30 s at a 15‐mA discharge current with an Ion Sputter (1010, Hitachi, Tokyo, Japan). The micrographs were obtained at an accelerating voltage of 2 kV.

### 2.10 RNA isolation and quantitative real time‐PCR

To perform qRT‐PCR, 3D‐bio larynx (*n* = 3) was cultured for 4 weeks in DMEM/F12 (free serum, 1% A/A). After 4 weeks of in vitro culture, only the hydrogel was used for analysis. Total RNA was isolated using TRIZOL reagent (Invitrogen, Carlsbad, CA, USA), following the manufacturer's instructions. Briefly, the hydrogels were placed in the TRIZOL reagent and homogenized using SuperFastPrep‐2™ (MP Biomedicals, Solon, OH, USA), followed by incubation at room temperature for 5 min. To remove the TRIZOL reagent, 100% chloroform was added to the collected supernatant and allowed to react at room temperature for 5 min. Only the uppermost layer (supernatant) was collected in a new tube after centrifugation for 15 min at 4°C and 13,000 rpm. Then, isopropanol was added to the tube and incubated for 10 min at room temperature, followed by centrifugation for 30 min at 4°C and 13,000 rpm. Subsequently, 70% ethanol was added to the collected pellet and centrifuged at 13,000 rpm for 5 min. After removing the ethanol, the tube was allowed to air‐dry for 1–2 h. Finally, 20 μl of diethyl pyrocarbonate (DEPC) water was added and held for 10–15 min in a 55–60°C water bath. qRT‐PCR was performed using LightCycler® 480 SYBR Green I Master (Roche Diagnostics, Mannheim, Germany) and primers (aggrecan, sox‐9, collagen type II, collagen type X, RUNX2, and MMP13). The quantified cDNA (50 ng) was used for real‐time PCR with a LightCycler® 408 Instrument II (Roche Diagnostics, Mannheim, Germany). Each sample was analyzed in triplicate and GAPDH was used as a reference. Fold‐change values were calculated based on Day 0. The PCR primer sequences are listed in Table 1.

**TABLE 1:** qRT‐PCR primers for cartilage specific gene expression analysis

Gene	Sequence (5′–3′)	Product size (bp)
GAPDH	F: CTCCTGCGACTTCAACAGT	175
	R: GTTTGAGGGCTCTTACTCCT	
ACAN	F: AGGTCTTGGAGTGACATCTG	232
	R: AGTAGAGCTGGACCACTGAA	
SOX‐9	F: CCTCTACTCCACCTTCACCT	198
	R: GTCACTTTGAGGCTCTTCTG	
Collagen type II	F: GGATAGACCCCAACCAAGGC	122
	R: GCTGCTCCACCAGTTCTTCT	
RUNX2	F: CCAAGTAGCCACCTATCACA	242
	R: TGGGGTCTGTAATCTGACTC	

Abbreviations: F, forward; R, reverse.

### 2.11 Animal study

This study was approved by the Institutional Review Board of Hallym University, Chuncheon, Korea (IRB No. 2016‐64). Six New Zealand rabbits (15‐ to 20‐week‐old, weighing 3.5–4.5 kg) were used in this study.

#### 2.11.1 Stage I: 3D‐bio larynx implantation into the greater omentum

The animals were anesthetized by intramuscular injection of a mixture of 2 ml of Ketamine® (Ketamine HCL, Huons, Gyeonggi, South Korea) and 1 ml of Rumpun® (xylazine chloride, Bayer Korea, Seoul, South Korea) at 0.5 ml kg^−1^ in a thigh region. The process of implantation of the 3D‐bio larynx into the omentum was as follows: after anesthetizing, each rabbit was placed in the supine position, and a 2‐cm‐long vertical incision was made at the midline of the abdomen. The omentum was identified through this incision and gently pulled out of the peritoneal cavity. The scaffold was wrapped in the omentum and sutured to the surrounding tissues using Nylon® 5‐0 (Johnson & Johnson, New Brunswick, NJ, USA). The abdominal muscles were approximated with 4‐0 Vicryl® (Johnson & Johnson) and the skin incision was closed with 4‐0 Nylon® (Johnson & Johnson) (Figure 2c).

#### 2.11.2 Stage II: 3D‐bio larynx implantation into the total laryngectomized model

Two weeks after implantation, the 3D‐bio larynx was harvested from the omentum (3D‐bio larynx‐OC) followed by transplantation into the laryngeal defect (Figure 2c) before implantation into the laryngeal defect. The newly formed connective tissue on the inner surface of the scaffold was removed, and connective tissue of the outer surface was preserved. A vertical skin incision was made at the midline of the neck, and the strap muscle was dissected into the anterior tracheal wall and larynx. Tracheostomy was performed to facilitate surgical procedures, and cuffed endotracheal tube (I.D. 3.5 mm/O.D. 5.3 mm/Cuff 8.0 mm, Teleflex®, Pennsylvania, USA), connected to ventilator (SV‐3000, SOAMED, Taipei, Taiwan) was placed into the tracheostoma to maintain general anesthesia (Sevoflurane®, Hana Pharm Co., Seoul, South Korea; flow rate 2 L/min). The exposed larynx was rotated to the contralateral side and the posterior border of the thyroid cartilage on each side was identified. The inferior pharyngeal constrictor muscle and the thyroid perichondrium were dissected from the thyroid ala using electrocautery. The lateral wall of the pyriform sinus was separated from the medial surface of the thyroid ala in a subperichondrial plane using a sponge and a freer elevator. During this procedure, both vocal folds were preserved. After incising the mucosa along the superior margin of the thyroid cartilage and inferior margin of the cricoid cartilage, the thyroid cartilage and cricoid cartilage were resected. The defect was replaced by the 3D‐bio larynx and sutured with Vicryl® 5‐0 (Johnson & Johnson, New Brunswick, NJ, USA). The anterior commissure of the vocal folds and epiglottis were repositioned at the 3D‐bio larynx with suture. A Levin tube (8FR; Sewon Medical Co, Ltd, Cheonan‐si, South Korea) was inserted, and a permanent tracheostoma was created.

### 2.12 Histological analysis

The specimens were embedded in paraffin blocks and sectioned into 4 μm‐thick slices. The slices were stained with hematoxylin and eosin (H&E), Masson's trichrome (MT), safranin O, and collagen type II. Endogenous peroxide was blocked with 3% H_2_O_2_ for 15 min. The sections were washed with PBS and blocked with horse serum for 1 h. The sections were washed with PBS and blocked with horse serum for 1 h. Then, they were incubated overnight with a collagen type II (diluted 1:200) primary polyclonal antibody (ab34712, Abcam, UK) raised in rabbits at 4°C. After washing with PBS, the sections were incubated with a goat anti‐rabbit secondary antibody (HRP, AbFrontier, Korea) for 2 h. After washing with PBS, the immunoreaction was visualized using diaminobenzidine (DAB; Sigma‐Aldrich) peroxide solution. Histological evaluation was performed to observe the formation of cartilage and collagen fibers using optical microscopy (Eclipse 80i; Nikon, Tokyo, Japan). The labeled collagen type II was observed using optical microscopy (Eclipse 80i, Nikon, Tokyo, Japan).

### 2.13 Statistical analysis

All data are presented as the mean ± standard deviation. Statistical analysis of experimental results was performed using GraphPad Prism 7 (GraphPad Software, San Diego, CA, USA). A *p* value was generated using Student's *t*‐test, with statistical significance set at not significant (ns), *p < 0.05, **p < 0.01, ***p < 0.001, and ****p < 0.0001.

## 3 RESULTS

### 3.1 ^1^H NMR spectroscopy

**FIGURE 4:**
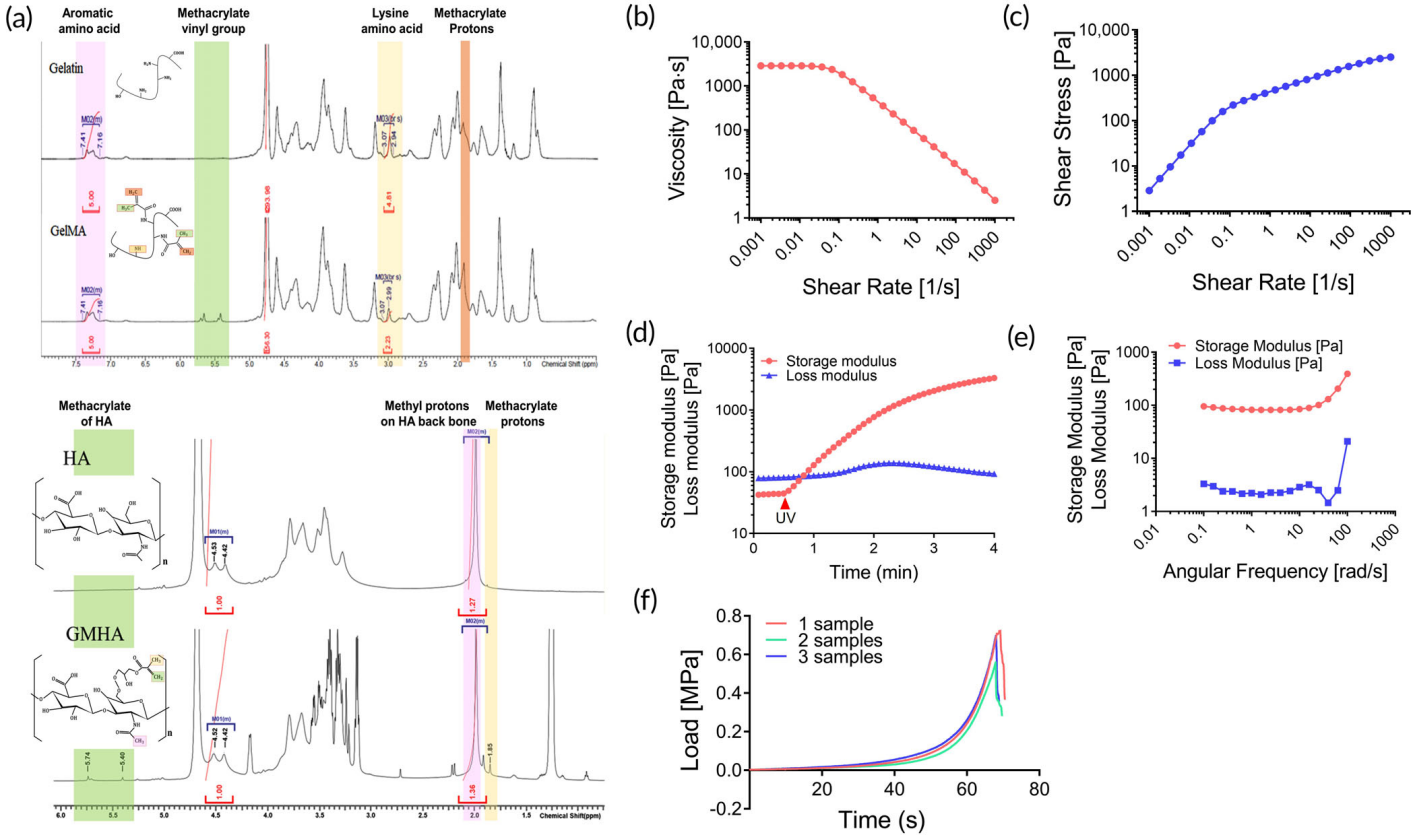
^1^H NMR, rheological and mechanical characterizations of the GelMA7%/GMHA5% hydrogel. (a) ^1^H NMR of methacrylated 2% gelatin and hyaluronic acid in D_2_O. (b) Viscosity, (c) shear stress, (d) storage modulus and loss modulus during UV exposure (the point at which storage modulus becomes greater than the loss modulus indicates gel point), (e) viscoelasticity, and (f) compressive strength

^1^ H‐NMR spectroscopy was performed to verify the methacrylation of gelatin and HA. Compared to the spectrum of unmodified gelatin, the GelMA sample showed new functional groups (Figure 4a). The peaks at around 5.3 and 5.5 ppm chemical shifts were assigned to the acrylic protons of the grafted methacryloyl group, and the peak at 1.9 ppm was attributed to the methyl group of the grafted methacryloyl group. The ^1^H NMR spectra of GMHA showed peaks at around 5.4–5.8 ppm, which were associated with the methacrylate group, and the peak at 1.85 ppm was related to the increase in methacrylate proton.

### 3.2 Rheological and mechanical properties of the bioink

We recently reported the rheological properties of the GelMA/GMHA bioinks, which were mixed in various ratios at 20, 24, and 30°C.^13^ Based on these results, G7H5 bioink was selected in this study, and we examined the rheological properties of the G7H5 bioink (Figure 4b–e). We also confirmed the compressive strengths of the G7H5 hydrogel, PCL, and PCL + G7H5 hydrogel constructs (Figure 4f). Our G7H5 bioink exhibited shear‐thinning behavior, indicating that the viscosity decreased as the shear rate increased, as shown in Figure 4b. Hydrogels with shear thinning, known as non‐Newtonian fluids, are suitable for extrusion‐based bioprinting.^14^, ^15^ In this regard, the G7H5 bioink was found to be well‐suited for our printing system in this study. The in situ storage modulus (*G*′) and loss modulus (*G*′) during UV exposure, in terms of the starting point for gelation, were also confirmed (Figure 4d). The compressive strength of the G7H5 hydrogel was 0.74 ± 0.063 MPa. The PCL scaffold had a compressive strength of 4.48 ± 0.214 MPa, six times that of the G7H5 hydrogel (*n* = 3; *p* < 0.0001). The PCL + G7H5 hydrogel scaffold had a compressive strength of 4.53 ± 0.246 MPa, and there was no statistical difference compared to the PCL scaffold.

### 3.3 FS system: Computational and in situ simulation analyses

Figure 3b shows the temporal concentration distributions of the glucose diffusion coefficient in the 3D‐bio larynx for 70 min. The aspect of concentration distributions was simulated, and the spatiotemporal variations in glucose were investigated for the entire domain every 5 min. As expected, glucose diffused from the dish to the 3D‐bio larynx over time. At 5 min, the medium showed slight glucose diffusion transfer through the immersed interface between 3D‐bio larynx and the culture medium domain. From the perspective view, the concentration of glucose around the 3D‐bio larynx becomes lower than that in most other places at high concentrations because the diffusive rate appears to be slightly small. As shown in the front view, the top region of the 3D‐bio larynx shows the minimum concentration of glucose, meaning that the diffusive transfer appears to be insufficient. However, the bottom regions corresponding to the immersed parts show a slightly higher concentration (15.2 mol/m^3^). At 35 min, near the 3D‐bio larynx boundary, the spatial distribution of glucose becomes apparently distinguished from the concentration distribution at 5 min. The maximum concentration at the corners of the dish decreased by approximately 13% (15.3 mol/m^3^), and the concentration distribution around the 3D‐bio larynx boundary shows wider spatial variations for 35 min. More glucose spread to the region in the absence of glucose, consequentially increasing the average concentrations around the 3D‐bio larynx boundary and within the structure. In the front view, the top region of the 3D‐bio larynx retains more glucose at a concentration of 14.36 mol/m^3^ and the bottom region at a concentration of 17.5 mol/m^3^. At 70 min, the distribution of glucose appeared to become more widely spread through the medium domain, and the concentration distributions around the 3D‐bio larynx boundary show wider spatial variations compared to the previous time frames and gradually flattened to a certain extent over time. In the front view, the top region of the 3D‐bio larynx attracts more glucose at a concentration of 15.27 mol/m^3^ and the bottom region at a concentration of 17.5 mol/m^3^, which determines a gradual gradient of concentration in the 3D‐printed larynx during 70 min. As the culture medium is released into the 3D‐bio larynx model, the concentration of glucose in the dish domain gradually decreases over time due to diffusion, and the diffusion rates are different for each time frame. Figure 3c shows the spatiotemporal variations in the glucose concentration along a hypothetical curve connecting points A and B within the 3D‐bio larynx, as indicated in Figure 3b. The initial concentration distribution along the line shows a minimum value, indicating that no glucose exists over the 3D‐bio larynx volume. Over time, diffusion occurs gradually into the 3D‐bio larynx. At 5 min, the concentration at point A near the medium domain rapidly increased, whereas no changes were detected at the top of the 3D‐bio larynx (point B). In the early stages, the distribution showed a steep gradient between the top and lower parts of the 3D‐bio larynx (along the hypothetical line). As time progressed, the molecules permeate the 3D‐bio larynx, and the gradient change became more gradual. The diffusion rates in the early stages appeared to be faster than those in the later stages, indicating that glucose was uniformly distributed over the 3D‐printed larynx volume. As indicated in Figure 3d, the temporal concentration distribution at the top of the 3D‐printed larynx (point B) showed almost no diffusive transfer up to the top during the first 5 min. The diffusion rate increased up to 30 min and gradually decreased as glucose accumulated in the upper parts of the 3D‐bio larynx. The adequate diffusion rate of the 3D‐bio larynx structure needs to be optimized by considering the consumption rate for feeding chondrocytes.

**FIGURE 5:**
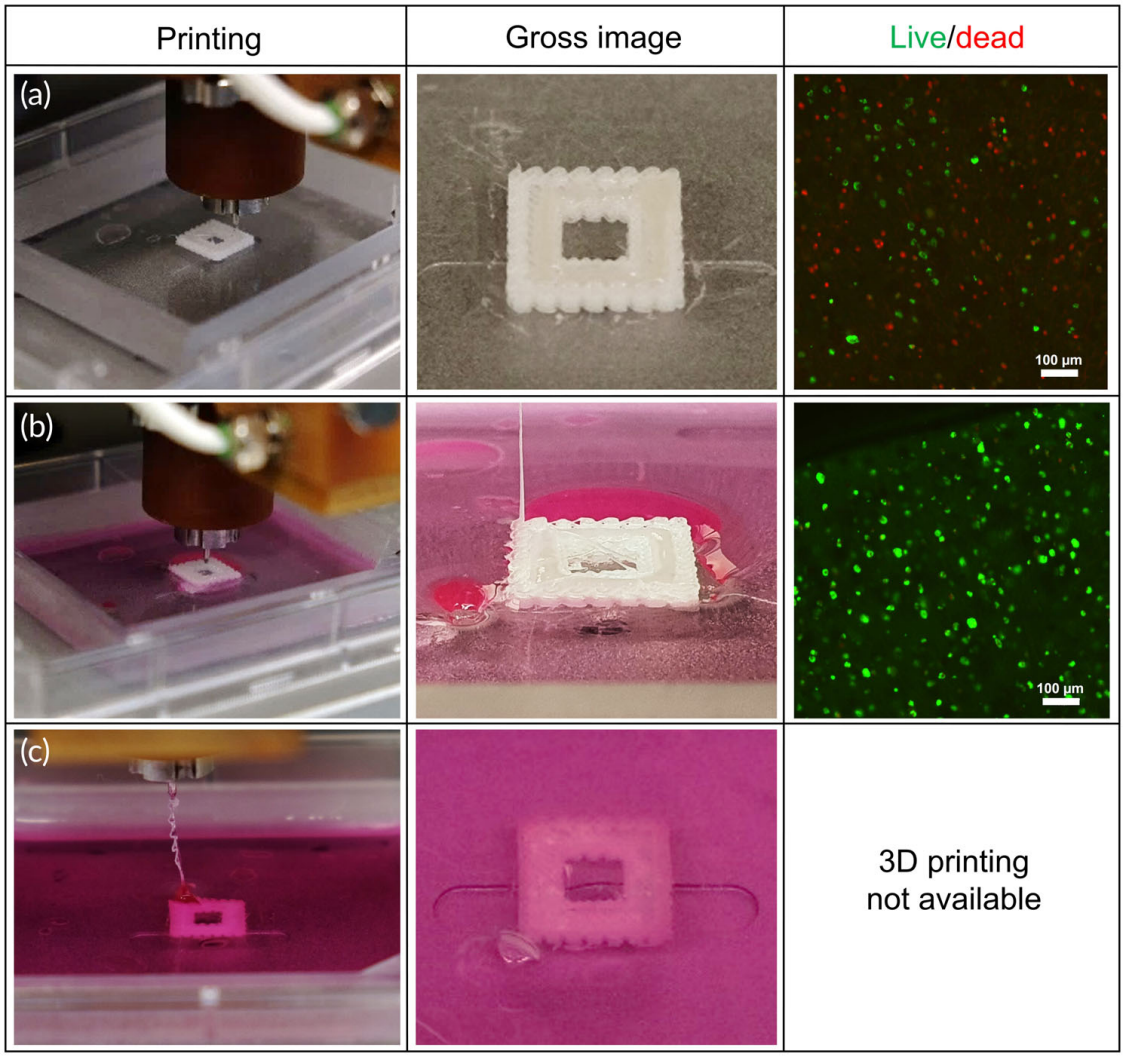
3D printing simulation with FS system. (a) Without fluid supply, (b) with moderate speed of fluid supply, and (c) with high speed of fluid supply

Figure 5 shows printability and cell viability with or without FS system. The upper panel demonstrated a 3D printed chondrocyte‐laden construct without the FS system. As expected, cell deaths were notified in live and dead assay without FS system. The middle and lower panels show printability and cell viability according to different media supplying speed. As indicated in Figure 5, excessive supplying speed compared to printing speed failed to generate a 3D construct (lower panel). On the other hand, adequate media supplying speed successfully generated a 3D construct with high cell viability. These results indicate that media supplying speed is one of the important factors in FS system. The media supplying speed should be modified depending on the printing speed. Cell viability with FS system was significantly higher than that without FS system (Figure S2), whereas it showed similar results in cell viability regardless of the type of supplying fluid. Supply PBS via FS system could also increase the cell viability. These results indicate that FS during printing could prevent drying out of the hydrogel, which had beneficial effects on post‐printing cell viability.

### 3.4 Cell proliferation test

The proliferation of chondrocytes gradually increased with time in both groups (Figure S1). Particularly, G7H5‐dissolved media showed higher cell proliferation than the control media (supernatant of DMEM/F12), which indicates that G7H5 bioink promoted more cell growth than DMEM/F12 (free serum, A/A 1%).

### 3.5 Effect of FS system on in situ cell viability

**FIGURE 6:**
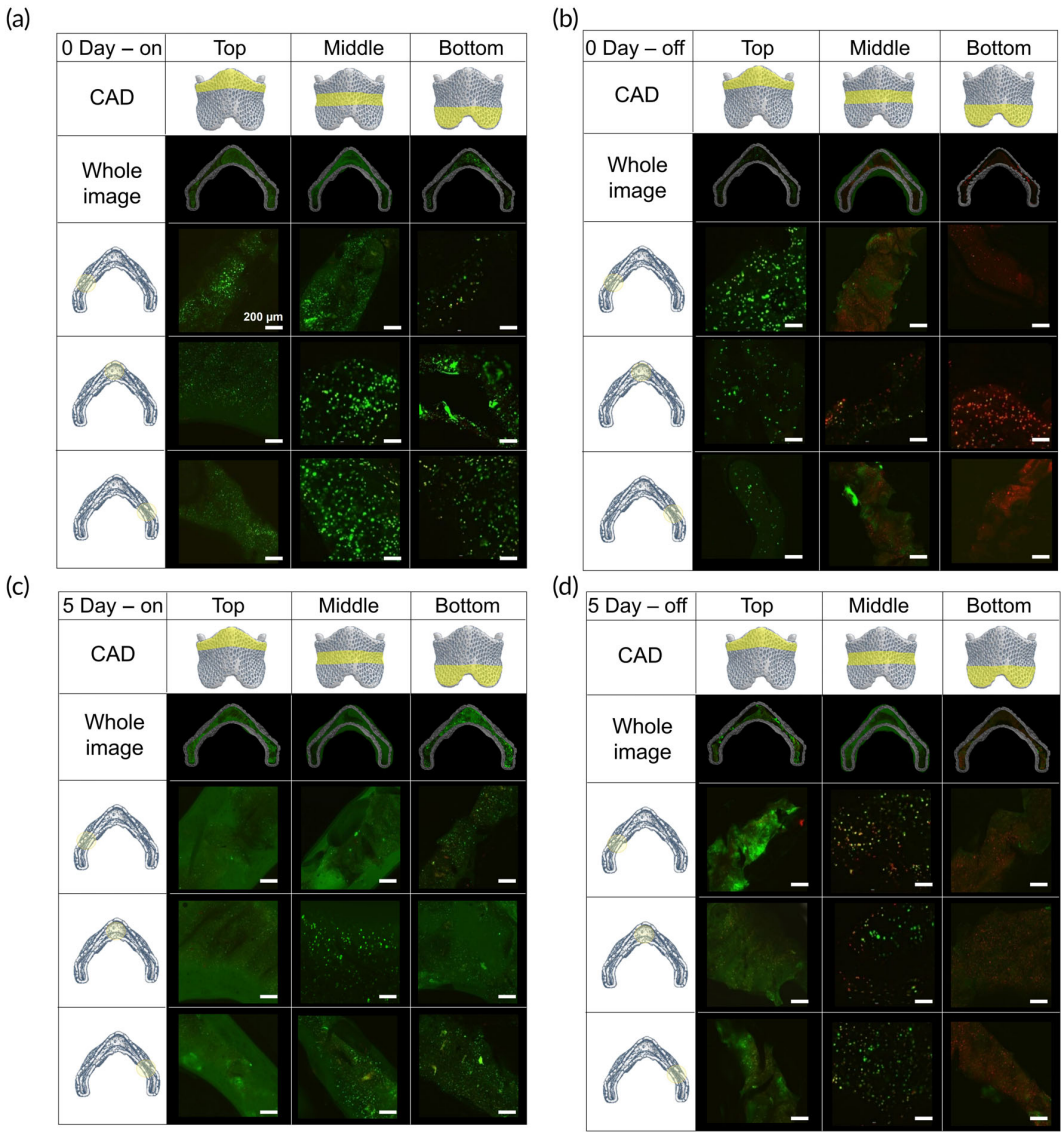
Effect of fluidics supply system on in situ cell viability. Cell viability immediately after 3D printing (a) with FS system and (b) without FS system. Cell viability of post‐5 days of 3D printing (c) with FS system and (d) without FS system. Scale bar: 200 μm

Chondrocytes were suspended in the G7H5 bioink, followed by 3D printing using an extrusion‐based 3D printer (3D Discovery Instrument, RegenHU Ltd. Inc., Villaz‐St.‐Pierre, Switzerland), with or without FS (Figure 6). The total printing time for the thyroid cartilage construct was 63 min. Cell viability was assessed at 1 h (0 day) and 5 days after 3D printing. 3D‐bioprinted thyroid cartilage constructs were cultured for 5 days in DMEM/F12 (free serum, A/A 1%). The chondrocyte‐laden construct was divided into three parts (top, middle, and bottom) to observe the effect of the FS system. As shown in Figure 6a–d, cell viability was significantly influenced by the installation of the FS system. In particular, the cell viability in the middle and bottom parts reduced significantly without the FS system. However, the cell viability of these parts increased 2‐ to 4‐fold after installing the FS system (Figure S3). This result was maintained for 5 days after printing and immediately after printing. The top part showed approximately 90% of cell viability regardless of the FS system. These results indicate that longer printing time caused higher cell damage. Specifically, in the bottom part, after an hour from the initial hydrogel printing, we could observe that nearly 80% of the cells died without the FS system. This result was attributed to the drying of the hydrogel during an hour of printing time. Figure S4 shows the temperature change in the printed PCL layer depending on the use of the FS system. The temperature of the printed PCL layer without the FS system was measured as 45–48°C; however, it decreased to 35–37°C when using the FS system. This result indicates that the thermal stress on the cells induced by the high temperature of the printed PCL layer reduces. Taken together, these results demonstrate that our FS system could prevent cell damage in all parts of the scaffold by preventing dehydration of the hydrogel as well as by minimizing thermal damage to the cells. All parts of the scaffold showed more than 90% post‐printing cell viability when using the FS system.

### 3.6 qRT‐PCR and morphology of the 3D printed chondrocytes: in vitro and in vivo

**FIGURE 7:**
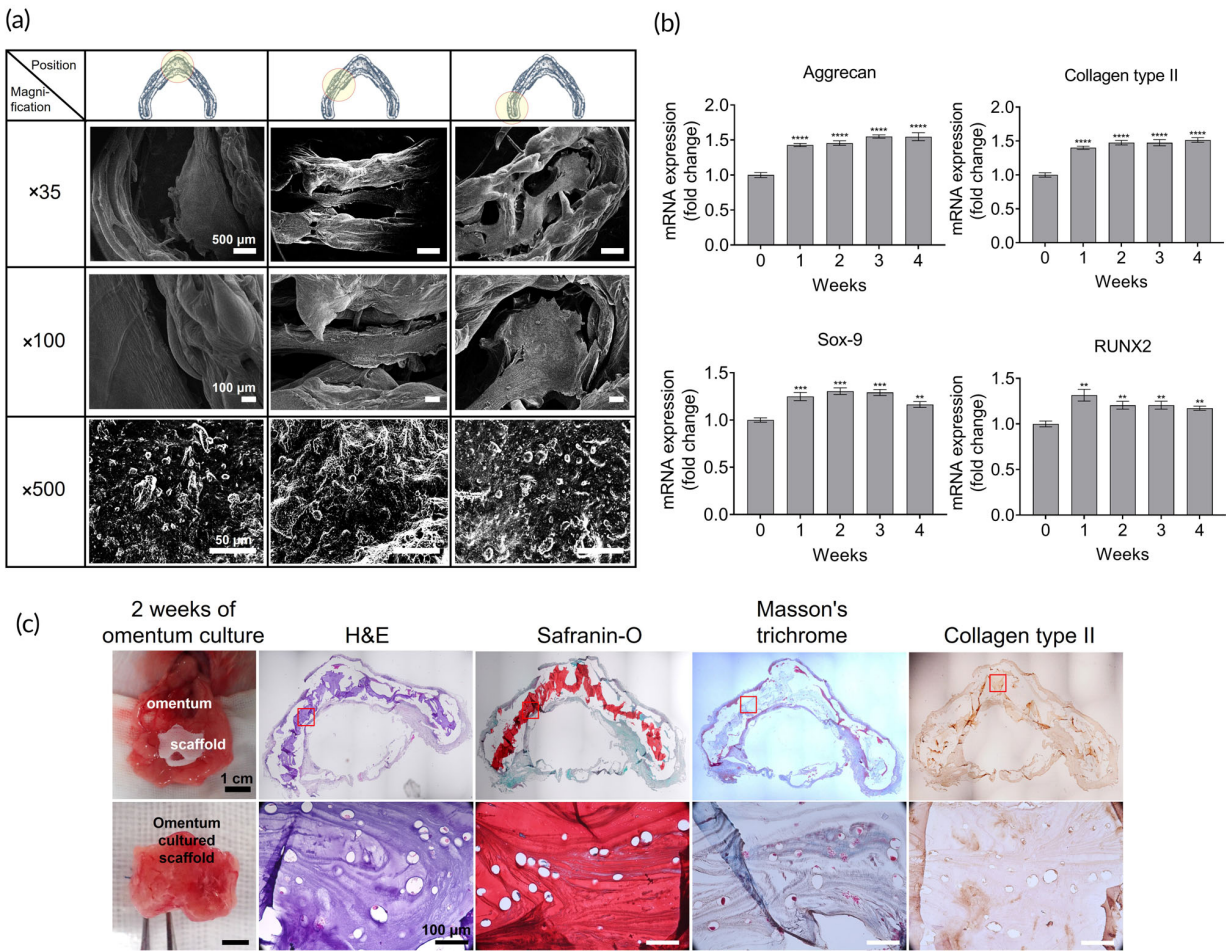
Morphology and qRT‐PCR of the 3D‐printed chondrocytes: in vitro and in vivo. (a) Representative scanning electron microscope images (at different magnifications) of the printed larynx construct cultured in DMEM/F12 (free serum, A/A 1%) for 2 weeks. (b) Expression of aggrecan and collagen type II was up‐regulated up to 4 weeks. SOX‐9 and RUNX2 expression was increased from 1 to 2 weeks, and then decreased. Expression of collagen type X and MMP 13 was gradually increased with time. (c) Histological images of the 3D‐printed construct after in vivo omentum culture for 2 weeks. H&E, hematoxylin and eosin

To observe the morphology of the printed cells, we used 2‐week‐cultured chondrocytes‐laden 3D‐bio larynx. As shown in the SEM results, chondrocytes presented a normal morphology with extensive cells present in the hydrogel (Figure 7a). The qRT‐PCR results for chondrocyte‐specific genes (aggrecan, SOX‐9, collagen type II, and RUNX2) are shown in Figure 7b. The expression of aggrecan and collagen type II was upregulated for up to 4 weeks. SOX‐9 and RUNX2 expression increased from 1 to 2 weeks and then decreased. These results indicate that the chondrocytes embedded in the G7H5 hydrogel maintained their physical properties for up to 4 weeks. SEM observations were further corroborated by histological analysis of the omentum‐cultured (for 2 weeks) scaffolds (Figure 7c). We observed chondrocytes embedded in the hydrogel using H&E and safranin O staining; Type II collagen expression was also observed.

### 3.7 Animal study

#### 3.7.1 Endoscopic findings

**FIGURE 8:**
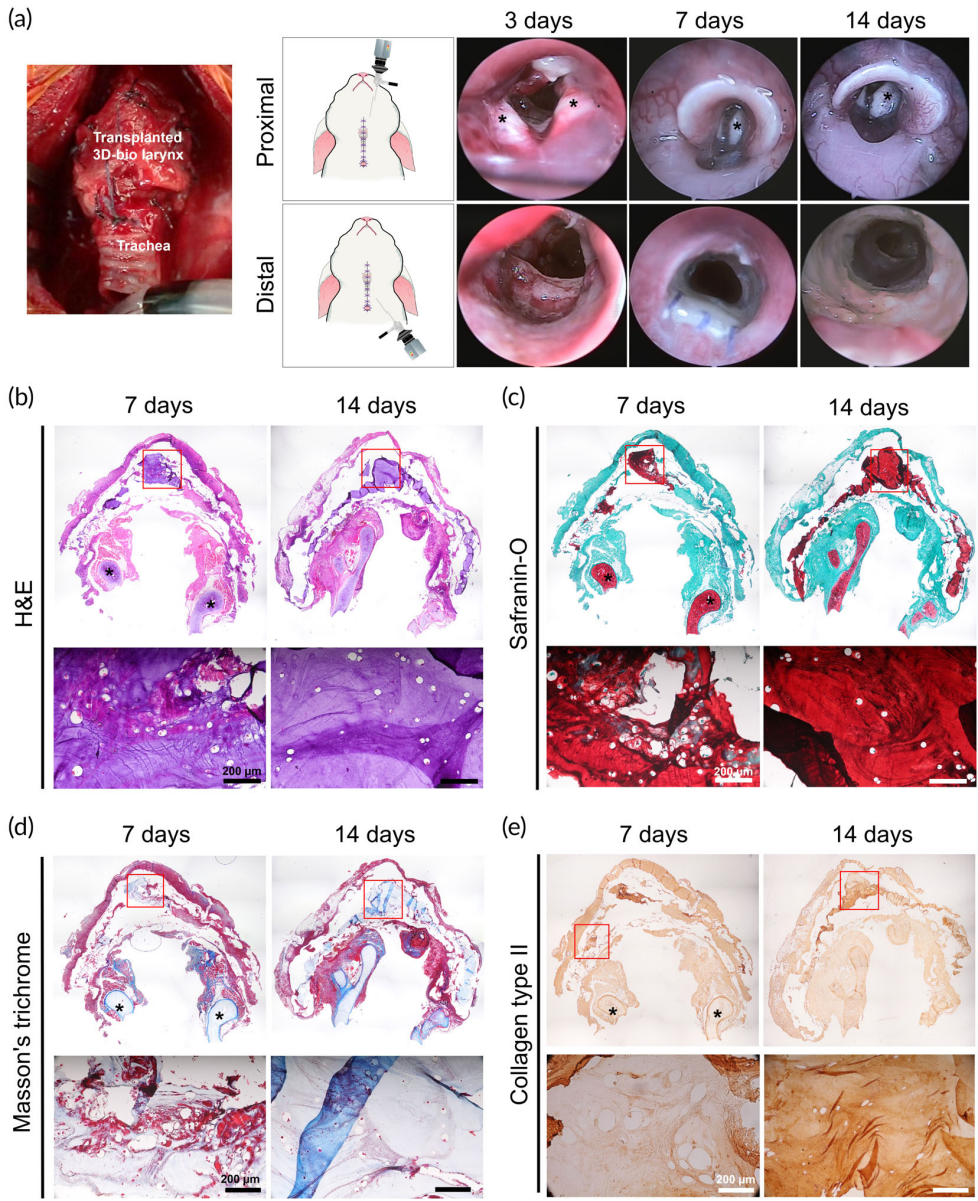
In vivo larynx transplantation. (a) Photographs of surgical procedures and endoscopic images of the transplantation area at post‐operative 3, 7, and 14 days, (b–e) histology of the 3D‐printed larynx after 1 and 2 weeks of in vivo transplantation. H&E, MT, safranin‐O staining, and immunohistochemistry for type II collagen. 3D bio‐larynx‐OC; 3D bio‐printed larynx after 2 weeks of omental culture. H&E, hematoxylin and eosin; MT, Mattson's trichrome

Endoscopic examination was performed to assess the scaffold transplantation area at 3, 7, and 14 days after transplantation (Figure 8a). From the endoscopic results, we observed that the epiglottis and the vocal cords were successfully reconstructed at the 3D‐bio larynx. They had a contour that structurally resembled normal anatomy. After passing through the vocal folds, the luminal surface of the 3D‐bio larynx was examined. The inner surface of the scaffold was covered with newly formed connective tissue. However, mucosal regeneration was not observed until 14 days after transplantation. The luminal diameter was well preserved without definite stenosis.

#### 3.7.2 Histology

As shown in Figure 8b–e, the 3D‐bio larynx maintained its structure until postoperative Day 14. In addition, the observation of chondrocytes printed in the G7H5 bioink revealed that chondrocytes maintained their normal morphology until 14 days post‐transplantation. The outer and inner surfaces of the 3D‐bio larynx were covered with newly formed connective tissue. In addition, we observed mild inflammation around the printed chondrocytes in the 1‐week specimen. However, inflammation decreased in the 2‐week specimen.

## 4 CONCLUSION

Laryngeal cancer is expected to account for 13,150 new cases and 3710 deaths in the United States in 2018.^16^ The most common surgical option for advanced laryngeal cancer is total laryngectomy, which negatively impacts the patient's quality of life profoundly, especially actions relating to swallowing, breathing, and speech.^17^ Although many approaches are available for laryngeal reconstruction after partial resection, including skin flap, myocutaneous flap, fascial flap, and thyroid gland flap, no satisfactory total laryngectomy replacement is available yet.^16^, ^17^, ^18^, ^19^, ^20^

Cells, one of the important elements of 3D bio‐printing, are sensitive to environmental changes; therefore, it is important to understand how 3D bio‐printing systems affect cells.^21^ In general, in situ cell viability is associated with the (1) biocompatibility of the bioink, (2) interaction of cell components with light, (3) thermal stress, and (4) mechanical stress during the printing process.^22^ In particular, microextrusion bio‐printing, which is used in this study, has some limitations such as low in situ cell viability resulting from shear stress in micro‐sized nozzles.^2^, ^4^, ^21^, ^23^, ^24^ In addition, a longer printing time causes low post‐printing cell viability owing to dehydration of the printed hydrogel. Several studies have suggested the use of biocompatible and mechanically stable macroscale scaffolds. Ragelle et al. suggested a surface‐tension‐assisted additive manufacturing method, which employs surface tension forces to coat reticulated supports with cell‐laden hydrogel.^9^ Yu et al. reported a reconfigurable microfluidic cell‐culture system that facilitates the assembly of 3D tissue models by stacking layers containing pre‐conditioned microenvironments.^10^ Further, the concept called cellular fluidics, suggested by Dudukovic et al., enables the creation of multiscale, cell‐based constructs with deterministic structure, porosity, and surface properties; therefore, this method has control over gas–liquid–solid interfaces and fluid flow.^11^

In this study, we presented an innovative method for enhancing post‐printing cell viability using an FS system. By installing the FS system, we successfully generated a large‐scale chondrocyte‐laden artificial larynx with more than 90% post‐printing cell viability. The FS system enhances the post‐printing cell viability via two mechanisms: (1) Our FS system prevents cell damage in all parts of the scaffold by preventing dehydration of the hydrogel, and (2) our FS system prevents thermal damage to the printed cells by decreasing the PCL layer temperature. In addition, our FS system can be installed with a simple design and equipment, and the fluid supply speed can be adjusted depending on the printing speed and the size of the printing bath.

To achieve structural strength of the printed constructs, we used a PCL outer framework incorporated with pores (200–500 μm). PCL is biocompatible, flexible, and more importantly, has a low melting temperature of 60°C to allow co‐printing with cell‐laden hydrogel.^25^ PCL has been proposed as a tracheal scaffold material in many studies, and our previous studies also confirmed its feasibility as a tracheal substitute because it maintained structural integrity with biocompatibility.^26^, ^27^ In this study, G7H5 hydrogel was selected to provide an optimal microenvironment for the printed chondrocytes. In our previous study, G7H5 bioink demonstrated excellent structural stability, including mechanical properties and printability, as well as reliable biocompatibility.^11^ In this study, G7H5 bioink was shown to provide a proper microenvironment for the 3D bioprinted chondrocyte. Cell proliferation tests showed that the G7H5 bioink stimulated more chondrocyte proliferation compared to control media. Aggrecan and collagen type II are major components of the extracellular matrix of hyaline cartilage, such as thyroid and cricoid cartilage,^28^ and qRT‐PCR data in this study demonstrated the promotion of the expression of these genes up to 4 weeks of in vitro culture. SOX‐9 is one of the earliest markers expressed in cells undergoing precartilaginous condensation, and RUNX2 is essential for chondrocyte maturation.^29^, ^30^ Taken together, these results indicate that the G7H5 bioink provides proper support to maintain chondrocyte innate with proper maturation.

In this study, we confirmed that a computer‐generated 3D PCL framework with pores successfully provided structural stability and facilitated nutrient transport to the chondrocyte‐laden hydrogel. We used 3D‐bio larynx cultured in DMEM/F12 or rabbit omentum for 2 weeks to evaluate in vitro and in vivo cell viability. We observed normal chondrocytes in the G7H5 bioink after 2 weeks of cell printing. These results revealed that the hydrogel parts could be directly connected to the basal media through pores in the PCL parts, and that nutritional support from the media could be sufficiently supplied to the cells in vitro.

To study whether the 3D‐bio larynx could replace the defective laryngeal framework, we transplanted 3D‐bio larynx into a total laryngectomized rabbit model. In our previous studies, we demonstrated the advantages of the omentum‐cultured trachea/esophageal scaffolds: (1) prior implantation of the scaffolds in the omentum is beneficial for revascularization of the scaffolds, (2) therefore, it leads to faster tissue regeneration and reduced associated complications such as scaffold exposure and stenosis at the anastomotic sites.^27^, ^31^ A major problem in engineered tissue is the cell death associated with in vivo transplantation.^32^ To address this problem, several solutions have been suggested to produce large‐scale cell‐laden constructs.^2^, ^22^, ^33^, ^34^, ^35^, ^36^, ^37^, ^38^ One possible solution is the pre‐vascularization of the scaffold in a bioreactor before transplantation, which can enhance the formation of vasculature within a 3D scaffold.^32^, ^38^ As shown in Figure 7c, prior omental implantation led to the formation of homogeneous, vascularized connective tissue on the surface of the 3D‐bio larynx.

In conclusion, the FS system can enhance post‐printing cell viability, enabling the generation of a large‐scale cell‐laden artificial laryngeal framework. Additionally, the incorporation of the PCL outer framework with pores and inner hydrogel provides structural stability and sufficient nutrient transport. However, our rabbit implantation study did not assess mature tissue regeneration because of the short experimental period. Long‐term and large animal studies are required to improve our understanding of tissue regeneration in the 3D‐bio larynx. Further, although we observed pre‐vascularization of the scaffold after omental implantation, methodologies for 3D‐printed larynx with dual functions of angiogenesis and chondrogenesis will need to be explored in a future study.

## AUTHOR CONTRIBUTIONS

**Hae Sang Park:** Conceptualization (equal); formal analysis (equal); investigation (equal); methodology (lead); visualization (equal); writing – original draft (lead); writing – review and editing (lead). **Ji Seung Lee:** Conceptualization (equal); data curation (equal); formal analysis (equal); investigation (equal); methodology (lead); writing – original draft (supporting); writing – review and editing (equal). **Chang‐Beom Kim:** Conceptualization (equal); data curation (equal); formal analysis (equal); investigation (equal). **Kwang‐Ho Lee:** Conceptualization (equal); formal analysis (equal); methodology (equal); writing – review and editing (equal). **In‐Sun Hong:** Conceptualization (supporting); validation (equal). **Harry Jung:** Formal analysis (supporting); investigation (equal); methodology (supporting). **Hanna Lee:** Investigation (supporting); resources (supporting). **Young Jin Lee:** Conceptualization (supporting); investigation (supporting); methodology (supporting); software (equal). **Olatunji Ajiteru:** Writing – review and editing (supporting). **Md Tipu Sultan:** Writing – review and editing (supporting). **Ok Joo Lee:** Project administration (lead); resources (supporting). **Soon Hee Kim:** Project administration (supporting); supervision (supporting). **Chan Hum Park:** Conceptualization (lead); funding acquisition (lead); supervision (lead).

## FUNDING INFORMATION

This work was supported by a National Research Foundation of South Korea (NRF) grant funded by the Korean government (MSIP; grant no. NRF‐2020R1A2C3010040), Republic of Korea, and the Hallym University Research Fund.

## CONFLICT OF INTEREST

The authors declare no conflict of interest.

### PEER REVIEW

The peer review history for this article is available at https://publons.com/publon/10.1002/btm2.10423.

## Supporting information


**Figure S1** Cell proliferation test.
**Figure S2**. Cell viability under different conditions of the FS system. FS system, fluidic supply system; On, with FS system; Off, without FS system; media, DMEM/F12 (free serum, A/A 1%).
**Figure S3**. Cell viability of the 3D‐bio larynx with or without the FS system. FS system, fluidics supply system; L, left; M, middle; R, right.
**Figure S4**. Temperature change on printed materials using FS system. FS system, fluidics supply system.

**Video S1** 3D printing process of the artificial larynx.

## Data Availability

The data that support the findings of this study are available from the corresponding author upon reasonable request.
