# Supplementary material for: Fluidic integrated 3D bioprinting system to sustain cell viability towards larynx fabrication
Source: Bioeng Transl Med. 2022 Oct 20;8(2):e10423. doi: 10.1002/btm2.10423 (PMC10013754; doi:10.1002/btm2.10423)
Supplement: Supplementary file 1 — Figure S1 Cell proliferation test. Figure S2. Cell viability under different conditions of the FS system. FS system, fluidic supply system; On, with FS system; Off, without FS system; media, DMEM/F12 (free serum, A/A 1%). Figure S3. Cell viability of the 3D‐bio larynx with or without the FS system. FS system, fluidics supply system; L, left; M, middle; R, right. Figure S4. Temperature change on printed materials using FS system. FS system, fluidics supply system. [file BTM2-8-e10423-s002.pdf]

Supplementary Information

Fluidic integrated 3D bioprinting system to sustain cell viability towards larynx fabrication.

Hae Sang Park[1,2,3*], Ji Seung Lee[2*], Chang-Beom Kim[4], Kwang-Ho Lee[5], In-Sun Hong[6], Harry Jung[3], Hanna Lee[2], Young Jin Lee[2], Olatunji Ajiteru[2], Md Tipu Sultan[2], Ok Joo Lee[2], Soon Hee Kim[2], Chan Hum Park[1,2]

[1]Department of Otorhinolaryngology–Head and Neck Surgery, Chuncheon Sacred Heart Hospital, College of Medicine, Hallym University, 77, Sakju-ro, Chuncheon, Gangwon-do 24253, Republic of Korea

[2]Nano-Bio Regenerative Medical Institute, School of Medicine, Hallym University, 1, Hallymdaehak-gil, Chuncheon, Gangwon-do, 24252, Republic of Korea

[3]Institute of New Frontier Research Team, Hallym University, Hallym Clinical and Translation Science Institute, 1, Hallymdaehak-gil, Chuncheon, Gangwon-do, 24252, Republic of Korea

[4]Intelligent Robot Research Team, Electronics and Telecommunications Research Institute, Daejeon 34129, Republic of Korea

[5]Department of Advanced Materials Science and Engineering, College of Engineering, Kangwon National University, Chuncheon, Gangwon-do, 24341, Republic of Korea

[6]Department of Molecular Medicine, School of Medicine, Gachon University, Incheon, 406-840, Republic of Korea

* These authors equally contributed to this work.

Corresponding Author

E-mail: hlpch@paran.com

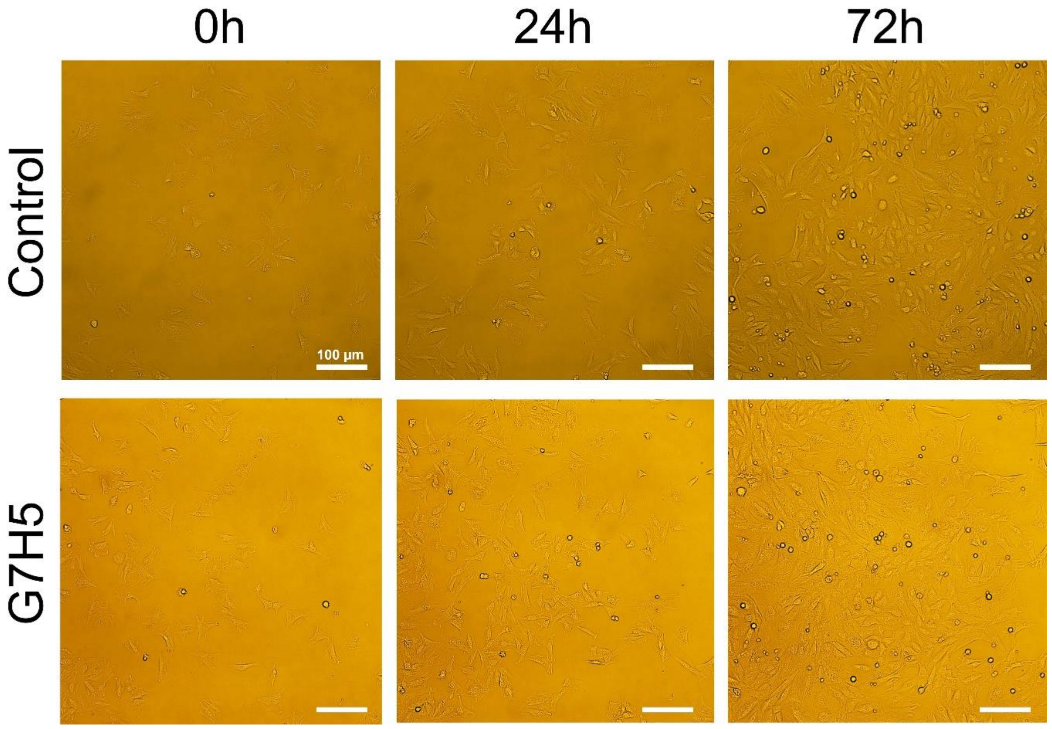

Supplementary Figure 1. Cell proliferation test

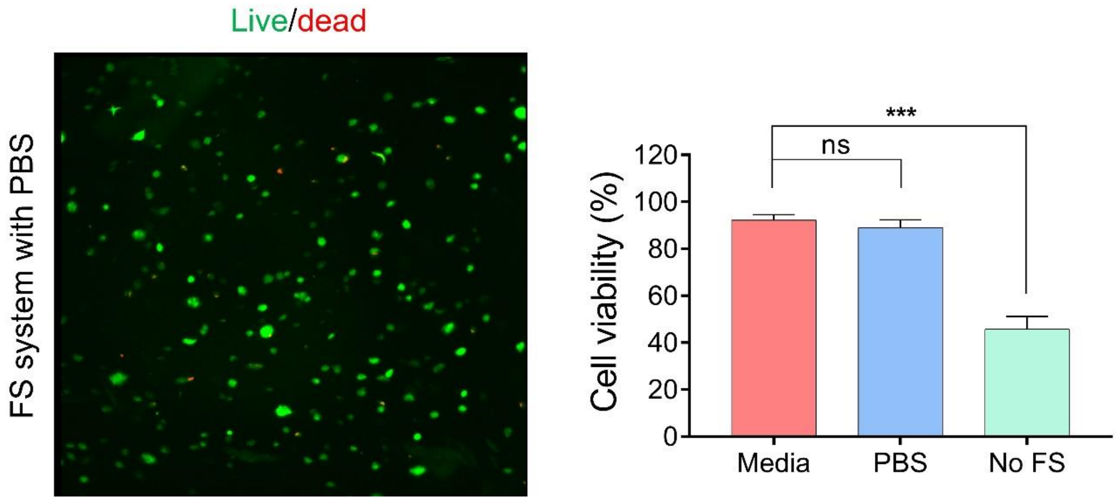

Supplementary Figure 2. Cell viability under the different conditions of the FS system. FS system, fluidics supply system; On, with FS system; Off, without FS system; media, DMEM/F12(free serum, A/A 1%).

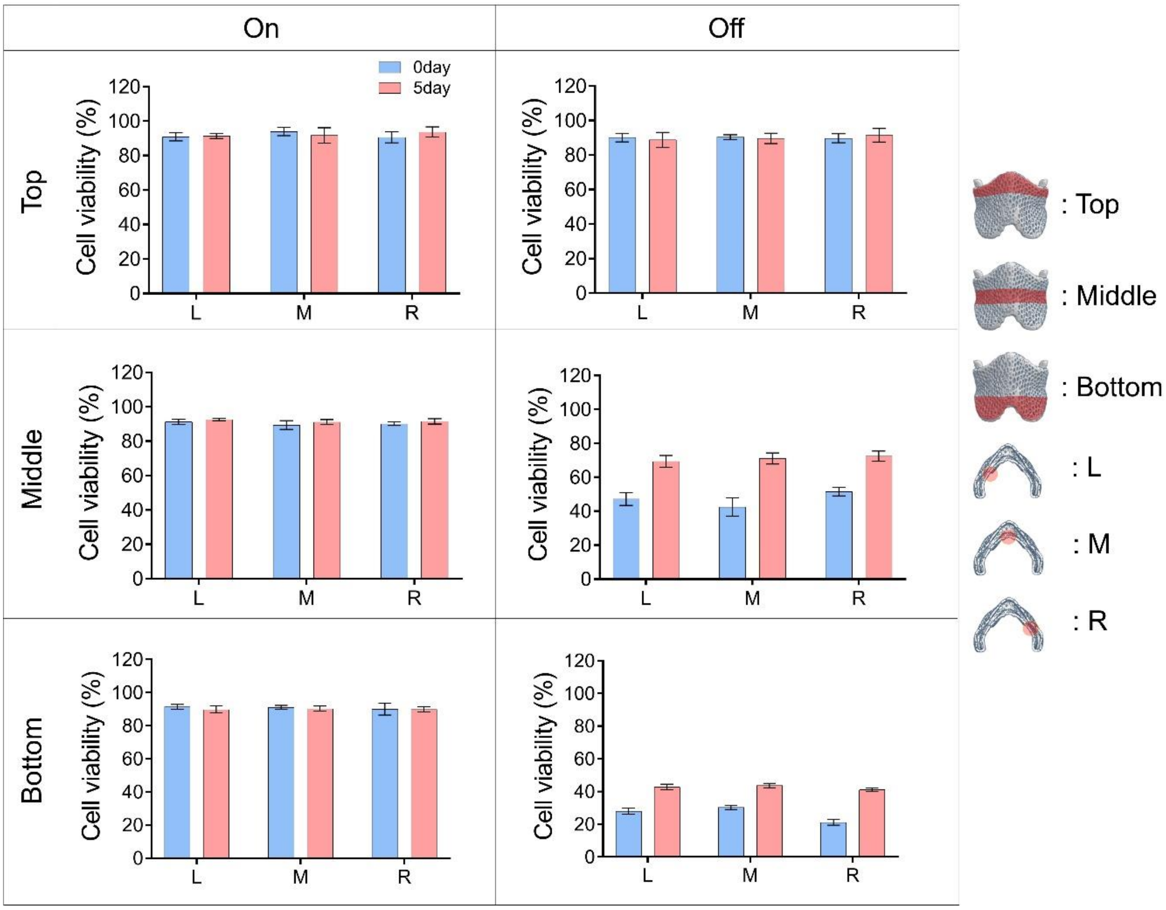

Supplementary Figure 3. Cell viability of the 3D-bio larynx with or without FS system. FS system, fluidics supply system; L, left; M, middle; R, right.

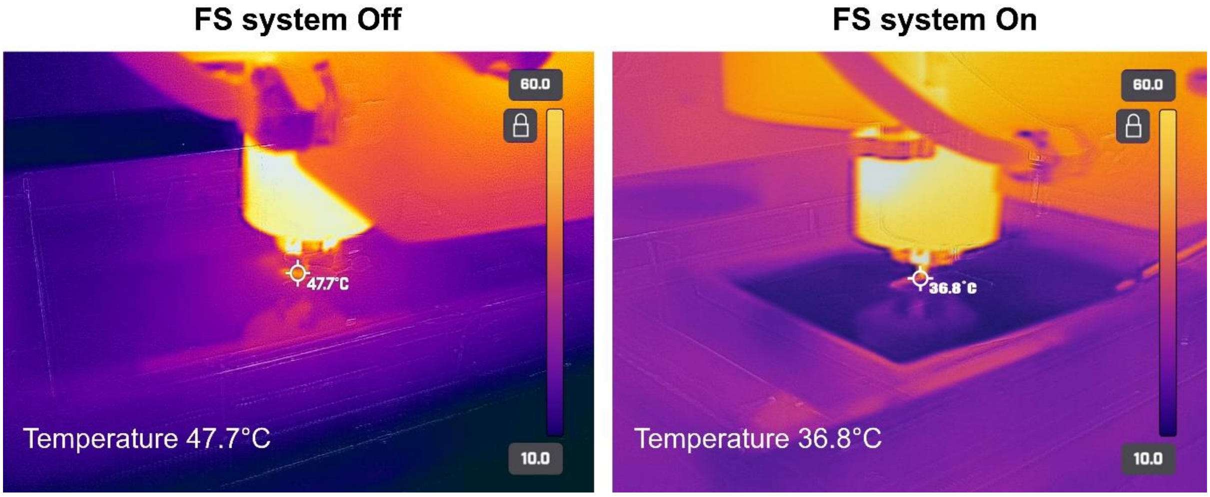

Supplementary Figure 4. Temperature change on printed materials using FS system. FS system, fluidics supply system.
